# Flavonoids and Flavonoid-Based Nanopharmaceuticals as Promising Therapeutic Strategies for Colorectal Cancer—An Updated Literature Review

**DOI:** 10.3390/ph18020231

**Published:** 2025-02-08

**Authors:** Andreea Smeu, Iasmina Marcovici, Cristina Adriana Dehelean, Stefania-Irina Dumitrel, Claudia Borza, Rodica Lighezan

**Affiliations:** 1 Faculty of Pharmacy, “Victor Babes” University of Medicine and Pharmacy Timisoara, Eftimie Murgu Square No. 2, 300041 Timisoara, Romania; 2 Research Center for Pharmaco-Toxicological Evaluations, Faculty of Pharmacy, “Victor Babes” University of Medicine and Pharmacy Timisoara, Eftimie Murgu Square No. 2, 300041 Timisoara, Romania; 3 Department of Functional Sciences, Discipline of Pathophysiology, “Victor Babes” University of Medicine and Pharmacy, 2 Eftimie Murgu Square, 300041 Timișoara, Romania; 4 Centre for Translational Research and Systems Medicine, “Victor Babes” University of Medicine and Pharmacy, 2 Eftimie Murgu Square, 300041 Timișoara, Romania; 5 Centre of Cognitive Research in Pathological Neuro-Psychiatry NEUROPSY-COG, “Victor Babes” University of Medicine and Pharmacy, 2 Eftimie Murgu Square, 300041 Timisoara, Romania; 6 Center for Diagnosis and Study of Parasitic Diseases, Department of Infectious Disease, “Victor Babes” University of Medicine and Pharmacy, 300041 Timisoara, Romania; 7 Discipline of Parasitology, Department of Infectious Diseases, “Victor Babes” University of Medicine and Pharmacy, 300041 Timisoara, Romania

**Keywords:** flavonoids, nanopharmaceuticals, colorectal cancer, therapy, targeted drug delivery

## Abstract

Colorectal cancer (CRC) represents one of the most serious health issues and the third most commonly diagnosed cancer worldwide. However, the treatment options for CRC are associated with adverse reactions, and in some cases, resistance can develop. Flavonoids have emerged as promising alternatives for CRC prevention and therapy due to their multitude of biological properties and ability to target distinct processes involved in CRC pathogenesis. Their innate disadvantageous properties (e.g., low solubility and stability, reduced bioavailability, and lack of tumor specificity) have delayed the potential inclusion of flavonoids in CRC treatment regimens but have hastened the design of nanopharmaceuticals comprising a flavonoid agent entrapped in a nanosized delivery platform that not only counteract these inconveniences but also provide an augmented therapeutic effect and an elevated safety profile by conferring a targeted action. Starting with a brief presentation of the pathological features of CRC and an overview of flavonoid classes, the present study comprehensively reviews the anti-CRC activity of different flavonoids from a mechanistic perspective while also portraying the latest discoveries made in the area of flavonoid-containing nanocarriers that have proved efficient in CRC management. This review concludes by showcasing future perspectives for the advancement of flavonoids and flavonoid-based nanopharmaceuticals in CRC research.

## 1. Introduction

CRC is a multifactorial malignancy that has become increasingly widespread around the world in recent decades, reaching up to 900,000 deaths annually [1]. Moreover, according to the latest estimation, the number of deaths caused by colon and rectal cancers will reach 71.5% and 60%, respectively, by the year 2035 [2]. As regards the risk factors for CRC, these are classified as modifiable (e.g., alcohol consumption, sedentary lifestyle, smoking, unhealthy diet, and mental stress) or non-modifiable (e.g., age, gender, genetic predisposition, family history, personal history of other diseases, and intestinal microbiota) [3]. The prognosis of the disease is poor, with a 5-year survival rate of approximately 10% for stage IV CRC and with many patients developing metastases, which are mostly incurable [4]. The currently known treatments (e.g., chemotherapy, targeted therapy, radiation, specific surgeries, cryosurgery, and radioimmunotherapy) are not efficient in all cases in curing the disease and also present severe toxic effects [3,5], urging research into therapeutic alternatives.

Recently, phytocompounds have attracted the attention of research studies as potential cancer treatments due to their more reduced side effects and the numerous beneficial biological activities that many classes possess. Flavonoids are natural products which consist of a large group of polyphenolic compounds ubiquitously distributed in plants and characterized by structure-dependent biological properties. The great interest in this class is attributed to the versatility of their activities, including hepatoprotective, antioxidant, anti-inflammatory, and antitumor effects [6], and their application in various therapies, particularly in cancer treatment, is receiving considerable recognition at present [6,7,8]. Flavonoids confer protection against tumor formation by either regulating specific signaling pathways involved in cell proliferation, apoptosis, and inflammation or by modulating the intestinal microbiota [8]. Many studies have described the nutritional value of flavonoids, and their consumption through diet has been closely linked to a reduced risk of CRC development [9]. In addition, flavonoids specifically target a variety of processes favoring uncontrolled CRC progress and spread, as they reduce DNA damage and mutational burden gain by minimizing oxidative stress, reduce inflammation by inhibiting pro-inflammatory pathways, trigger cancer cell death by altering the cell cycle and activating apoptosis, and limit tumor blood supply by suppressing angiogenesis [2]. However, despite these therapeutic benefits, flavonoids are also characterized by several limitations, such as reduced selectivity, low solubility in water, inadequate gastrointestinal absorption, and rapid metabolism, that hamper their therapeutic applications and need to be addressed [6,10,11].

Nanotechnology, one of the most promising technologies of the 21st century, which refers to the measurement, control, assembly, and manufacture of matter at the nanoscale level, has emerged as a revolutionary solution for cancer diagnosis and treatment [12,13]. This is especially due to the breakthrough and design of nanopharmaceuticals, comprising a pharmaceutical agent entrapped in a nanoscaled carrier (e.g., liposomes, nanogels, nanoparticles, microemulsions, and polymeric micelles), which have become the latest nanotechnological advancement to improve the solubility, pharmacokinetics, release profile, target specificity, and safety of the delivered compounds [14,15,16]. The inclusion of nanopharmaceuticals in chemotherapeutic regimens arises as a novel and efficient treatment for different cancer types, conferring numerous advantages in comparison with traditional therapies, such as increased bioavailability, better cell internalization and trans-membrane penetrability, reduced side effects, and selective drug unloading in the targeted region [17,18]. In particular, nanomedicine in the area of CRC research has substantially expanded, especially in countries such as China, the United States, and India, according to a 12-year bibliometric and visual analytics study [19]. Additionally, a vast array of preclinical studies support the inclusion of chemotherapy-based nanoformulations in CRC treatment regimens, with some, although their number is still limited, reaching the stage of clinical trials, as comprehensively reviewed recently [20]. The study of nanoformulated phytochemicals has attracted great interest lately for the potential treatment of CRC, considering that nanotechnology might minimize some of their limitations, while concomitantly potentiating their biological activity. The methods by which nanoscale formulations can be combined with natural compounds are manifold, but among them can be listed thin film hydratation, high-pressure homogenization, nanoprecipitation, or self-assembly [21,22]. The therapeutic activities of plant extracts are mostly attributed to the phytochemical contribution, usually associated with the presence of flavonoids, alkaloids, polyphenols and others. Therefore, green synthesis is also being used as an ecological method to support the generation of nanostructures (e.g., silver nanoparticles) with phytochemical substances [23].

In light of these aspects, the current paper outlines the most recent advances in the study of flavonoids and their nanopharmaceuticals as potential therapeutic strategies targeting CRC. Debuting with a presentation of the pathologic features of CRC and the limitations of the existing treatments, the review mechanistically describes the pharmacologic effects of flavonoids and underlines the signs of progress made in their nanoformulation using targeted delivery systems as a promising next-generation option for CRC management.

## 2. General Aspects on CRC

CRC is the third most frequently diagnosed cancer worldwide, being more common in women, and the second most prevalent neoplasm in North America, Western Europe and Oceania [24]. Although the risk increases after the age of 45, with the highest incidence and mortality rates being encountered at the age of 80 [24], advanced stages of CRC are also diagnosed in young people [25]. Recent statistics reported that approximately 153,000 people were diagnosed with CRC and approximately 52,550 died from CRC in 2023. Despite the progress made, CRC is starting to appear in the diagnosis of young people at more advanced stages [25] and also, according to another estimation, approximately 106,590 and 46,220 new cases of colon cancer and rectal cancer, respectively, were expected in 2024 [26]. Approximately 90% of CRCs are adenocarcinomas, but there are other less common types such as carcinoid tumors, colorectal lymphoma or squamous cell carcinomas, with more than half developing without a family history or predisposition to inherited genetic mutations [4].

Risk factors are varied and can include smoking, obesity, lack of physical activity or improper diet. The main sites of CRC metastasis are the liver and peritoneum, with peritoneal carcinomatosis being considered the final stage after the tumor has spread to the liver [27]. From a histological perspective, CRC gradually develops by navigating through distinct stages, as follows: (i) Stage 0—polyps formation within the inner space of the colon’s lining of epithelial cells, (ii) Stage I—polyps transformation into tumors and invasion into the inner colon mucosa, (iii) Stage II—tumor expansion into the external layer of the colon by without reaching of the lymph nodes, (iii) Stage III—tumor complete penetration of the colon walls and spreading to the lymph nodes, and (iv) Stage IV—metastasis to distant organs [18].

Screening and early detection of localized cancer and precursor lesions are among the greatest opportunities to reduce the burden of the disease, with colonoscopy being acknowledged as the most popular diagnosis method [28,29]. Current treatments for CRC include surgery, preoperative radiotherapy, systemic therapy, local ablative therapies, palliative chemotherapy, cryotherapy and immunotherapy [1,30]. However, conventional anticancer treatments present numerous limitations, including multiple side effects such as anemia, thrombocytopenia, vomiting, infections, neutropenia, and decreased sleep quality [31]. Furthermore, there may also be a risk of recurrence or drug resistance, a common problem for oncological patients [32], hastening the continuous research of new treatment options.

## 3. General Overview on Flavonoids

### 3.1. Therapeutic Properties of Flavonoids

Flavonoids are the most widespread phenolic compounds found in plants, distributed in both fruits and vegetables [33], and containing a basic structural unit of 2-phenylchromone [34]. Apples, citrus fruits, berries, onions, broccoli and kale are the principal sources of flavonoids, although tea and wine are also important flavonoid reservoirs [35]. At present, approximately 5000 flavonoids have been identified and separated from different sources [34]. Flavonoids are part of the human diet as well, being consumed daily at approximately 400 mg/kg aglycone equivalent [33]. Flavonoids are hydroxylated phenolic substances synthesized by plants via the phenylpropanoid pathway for multiple purposes, including as a defense against microorganisms [36], and to protect plants from exposure to atmospheric UV light stress, but also contributing to flower coloration and flavoring [37].

Apart from their benefits in planta, flavonoids are also characterized by low adverse reactions compared to conventional treatments and numerous pharmacological properties (e.g., anticancer, anti-inflammatory, antioxidant, protective or antiviral) that have brought these natural products into the spotlight of the current research for potential medical-pharmaceutical applications [34]. This class of natural compounds has brought substantial benefits for the management of pathologies that represent serious global health issues (e.g., Alzheimer’s disease, epilepsy, hypertension, diabetes mellitus, hearing affections, lung affections, bone affections, and viral diseases) [38,39,40,41,42,43,44]. It has also been reported that some flavonoids possess anti-fungal potential against *Fusarium brachygibbosum*, *Aspergillus niger* and *Fusarium avenaceum* [45]. Additionally, it was observed that flavonoids have the ability to reduce the cyclooxygenase and lipoxygenase activity, platelet aggregation, fragility and capillary permeability, but also block a variety of enzymes such as hydrolases, hyaluronidase, alkaline phosphatase, arylsulfatase or cAMP phosphodiesterase [46]. Furthermore, flavonoids are also promising candidates in anticancer therapies [47]. European studies in the nutritional field have shown that the daily intake of flavonoids in different ethnic populations is related to the quality of health, especially concerning cancer, which marks the importance of these compounds given the poor prognosis of cancers and increased risk of occurrence [48]. The effects in the oncological area are currently being studied extensively because flavonoids have proven their efficacy against several types of cancers, including colorectal, breast, lung, skin, prostate and bladder cancers [49]. Specifically, flavonoids exhibit a variety of biological effects that may cover several functions involved in cancer development, by producing reactive oxygen species (ROS), induce apoptotic cell death, autophagy, participate in cell cycle arrest and suppress cell growth, proliferation and invasion [50]. Inhibition of angiogenesis is also an important function of flavonoids in cancer therapy, as it leads to the invasion of cancer cells [51].

### 3.2. Classification of Flavonoids

**Figure 1:**
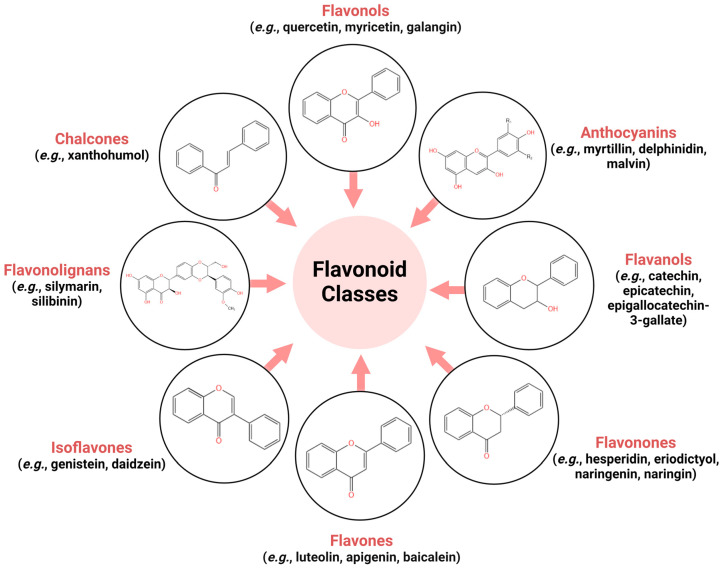
Schematic overview of the major flavonoid classes and the representative compounds belonging to each class. This image was created using Biorender.com and KingDraw.com (accesed on 15 January 2025).

The biological activities of flavonoids are structure dependent, while their structural aspect depends on the chemical class, hydroxylation grade, polymerization grade, and other conjugations or substitutions. Flavonoids are chemically composed of a skeleton of fifteen carbon atoms consisting of two benzene rings linked by a dihydro-pyran ring, and can thereby be divided into a variety of classes. Flavonoid classes differ in terms of oxidation level and substitution pattern of the dihydro-pyran ring, and individual compounds within a class vary in terms of the substitution pattern of the benzene rings [6]. Based on structural differences, flavonoids are divided into multiple classes: flavonols, flavones, isoflavones, anthocyanins, flavonones, flavanols, chalcones, flavolignans and isoflavones [36,52]. A schematic overview of the main flavonoid classes and the representative compounds belonging to each class is provided in Figure 1.

Flavonols are well-recognized compounds (e.g., quercetin, myricetin, and galangin) that possess hydroxyl groups at positions 5 and 7 of the A-ring and hold multiple important biological activities such as anticancer, anti-inflammatory, antioxidant or antibacterial. Consumption of flavonols has been reported as protective against gastric cancer in women and smokers [53]. Flavones (e.g., luteolin, apigenin, and baicalein) are a group of flavonoids that possess the double bond C2′-C3′ and the C-ring, which carries a ketone at the C4′ position. A large proportion of flavones have an A ring, which bears a hydroxyl group at the C5′ position, with hydroxylation normally occurring at the C7′ position of the A-ring and at the C3′ and C4′ positions of the B-ring. However, the glycosylation of flavones predominantly takes place at the C5′ and C7′ positions of the B-ring, and their methylation and acylation occur in the B-ring of the hydroxyl groups. They are found in a panel of leaves, flowers and fruits [54]. Isoflavones (e.g., genistein and daidzein) are prevalent in legumes and have been of interest to researchers in recent decades for their health benefits. The chemical structure comprises a 3-phenylchromen-4-one backbone, with the rings labeled A, C and B, starting from the left [55]. Anthocyanins (e.g., myrtillin, delphinidin, and malvin) may be found in many fruits and vegetables and are often used as natural colorants. They have innumerable biological effects including antimicrobial properties, antioxidants, and protective against the progression of cardiovascular diseases, neurodegenerative illnesses and cancers [56]. Flavonones or dihydro-flavones display a saturated C-ring. They are distributed in citrus fruits and, depending on their chemical structure, include agents such as hesperidin, eriodictyol, naringenin or naringin [53]. Chalcones, with their representative xanthohumol, are precursors of flavonoids and isoflavonoids, their structural characteristics being easily built from simple aromatic compounds. Their pharmacological properties comprise of a wide range of effects, these molecules showing antioxidant, anthelmintic, antiulcerative or antitumoral effects [53]. Another subgroup of flavonoids are the flavanole (e.g., catechin and epicatechin) and in the same way as the other subgroups, they possess many health-promoting properties such as anti-inflammatory or anti-infectious, anticancer, antioxidant, or vasculo-protective. They are also recognized for their benefits in the management of Alzheimer’s or Parkinson’s disease, but also in cardiovascular conditions [57]. Flavonolignans are compounds with potent anticarcinogenic effects and the main active components of silymarin extracted from the species *Silybum marianum* L. [58]. Silymarin is known for its numerous biological activities including anti-inflammatory, antiproliferative, lipid antiperoxidative and antifibrotic. As regards cancer treatment, studies have revealed that it acts upon various processes such as cell cycle, metastasis, angiogenesis, autophagy and apoptosis [59].

### 3.3. Delivery Systems for the Development of Flavonoid Nanopharmaceuticals

Drug delivery systems represent a variety of platforms that are designed to direct and release compounds to a defined target, such as specific cells, tissues or organs. Since the 1950s, modern drug delivery systems have made outstanding progress, with over 100 nanomedical products being approved by the Food and Drug Administration (FDA) [60,61]. Nanopharmaceuticals are defined as pharmaceutical products manufactured in a nanosized form, in which the nanomaterial performs the essential therapeutic role or provides superior functional properties to the encapsulated compound [62]. These have demonstrated safety and efficacy, improved permeation through biological barriers, and high ability for the delivery and accumulation of drugs to malignant tumors [61].

Despite the numerous health-promoting benefits exerted by flavonoids (e.g., anticancer, antioxidant, antimicrobial, and anti-inflammatory), they present several disadvantages that delay their utilization in practice. The low water solubility of flavonoids is among the best-known barriers that can reduce the oral bioavailability of these compounds [63]. Poor absorption is also a strong limitation for the therapeutic use of flavonoids [64], while studies have also mentioned the reduced chemical stability of some compounds belonging to this class (e.g., baicalein and myricetin) [65,66]. Often, only a fraction of the administered compound reaches the systemic circulation and the target organs. Similar difficulties arise with topical application, where the skin barrier needs to be crossed. Preclinical studies have indicated that the internal organs have no direct contact with flavonoid aglycones, but only with their metabolites; therefore, the doses of aglycones used in in vivo experiments were never reached at the desired level in the body [67]. Further, a recent paper has specified that oral administration may affect the bioavailability of flavonoids not only due to their solubility limitations, but also due to the chemical modifications caused by the intestinal microbiota [68]. Furthermore, one study also mentioned the need for nanoencapsulations to avoid the gastrointestinal degradation of flavonoids by fluids [69].

Nanoformulation approaches for encapsulation of flavonoid-derived compounds are a promising option for protecting them from external factors [70] and, moreover, many papers have also demonstrated that nanocarriers can improve the biological potency of compounds in various diseases [67]. This strategy also brings advantages in terms of topical administration, the most suitable nanoencapsulations for this type of administration being liposomes, hydrogels, nanofibers, solid lipid nanoparticles or polymeric micelles that confer specific delivery and improve bioavailability and solubility, but also protect flavonoids from degradation [69].

**Figure 2:**
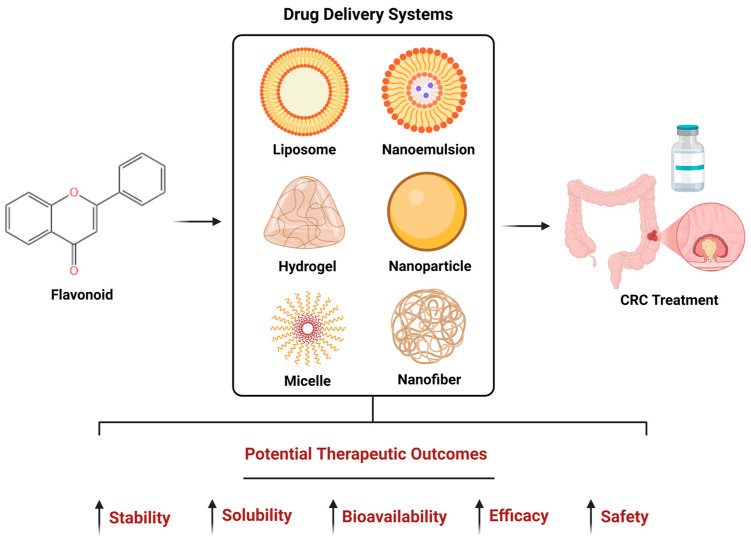
The formulation of flavonoids as nanopharmaceuticals through incorporation in different drug delivery systems might result in potentially improved outcomes in the treatment of colorectal cancer (CRC). This image was created using Biorender.com (accesed on 15 January 2025). ↑ increased.

To improve bioactivity and therapeutic effects, flavonoids have presently been studied in several types of nanoformulations, such as nanoemulsions, chitosan nanoparticles, copper nanoparticles, iron nanoparticles or liposomes for which bioavailability enhancement, association with other agents, improvement of flavonoid efficacy or transport to the target were explored [71]. In addition, the increase in the permeability (via lipid-based nanoparticles, nanoemulsions, liposomes), solubility (via polymeric nanoparticles), selectivity (via lipid-based nanoparticles), safety and overall stability (via liposomes, niosomes) of flavonoids are other strengths that certain nanosystems can provide [72]. Flavonoids are low soluble in water due to their hydrophobicity (e.g., for quercetin 0.01 mg/mL, genistein 0.01 mg/mL and luteolin 0.0064 mg mL^−1^), which is dependent on the number of hydroxyl groups [73,74,75,76]. The use of nanoparticles is an efficient method in the advancement of drug delivery with low solubility because nanoparticles possess a high specific surface area, which consequently leads to increased dissolution rate and solubility of the entrapped compounds [77]. Some flavonoids (specifically quercetin and luteolin) were found to be sensitive to atmospheric oxygen, which was shown to cause their degradation and decrease their potential pharmacological properties [78]. In terms of absorption problems, some flavonoids were considered to be non-absorbable because they are bound to sugars in the form of beta-glycosides, except aglycones that are able to pass through the intestinal wall [6,79]. As a potential solution, nanocarriers generally have high dispersion characteristics that help achieve an optimized binding between the drug and the absorption site [80]. Also, the shape, size and surface area of nanoparticles can have a substantial impact on cellular uptake and efficacy, as those with smaller sizes (50–200 nm) are more easily transported through intestinal cells compared to larger ones [81]. Flavonoids also might suffer sulfation, methylation and glucuronidation in the small intestine and the liver resulting in conjugates with reduced bioactivity compared to parent compounds [79]. Related to this, nanocarriers might confer protection to flavonoid compounds from premature degradation [71]. An overview on the potential outcomes resulting from the incorporation of flavonoids in different drug delivery systems is presented in Figure 2.

## 4. Flavonoids and Flavonoid-Based Nanopharmaceuticals for CRC Treatment

### 4.1. Flavonoids as Potential Therapeutics for CRC

**Figure 3:**
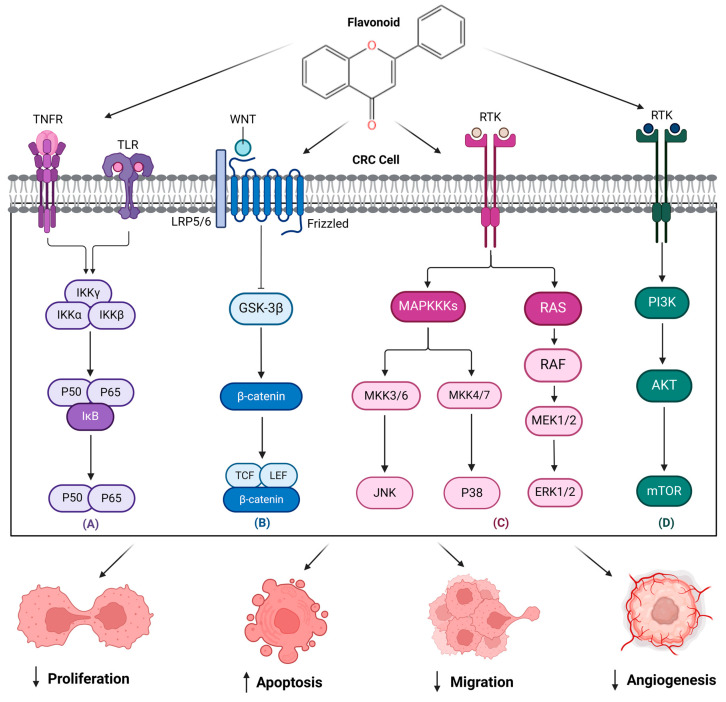
Flavonoids target several pathways, i.e., NF-kB—(**A**), Wnt/β-catenin (**B**), MAPK (**C**), and PI3K/AKT/mTOR (**D**), involved in CRC development and progression. This image was created using Biorender.com and KingDraw.com (accessed on 15 January 2025). CRC—colorectal cancer; NF-kB—nuclear factor-kappa B; Wnt/β-catenin—the canonical Wnt pathway; MAPK—Mitogen-activated protein kinase; PI3K/AKT/mTOR—phosphoinositide 3 kinase (PI3K)/Akt/mammalian (or mechanistic) target of rapamycin (mTOR); TNFR—tumor necrosis factor receptors; TLR—toll-like receptor; RTK—receptor tyrosine kinase; IKK-γ—inhibitor of κB kinase; IKK-α—inhibitor of nuclear factor kappa-B kinase subunit alpha; IKK-β—inhibitor of nuclear factor kappa-B kinase subunit beta; P50—protein P50; P65—protein P65; IkB—IkappaB kinase; LRP6/6—low-density lipoprotein-related receptors 5 and 6; GSK-3B—glycogen synthase kinase 3 beta; TCF/LEF- transcription factors; MAPKKK—Mitogen Activated Protein (MAP) kinase kinase kinase (MAPKKK, MKKK, M3K, or, MAP3K); JNK—Jun N-terminal kinase; P38—protein P38; MEK ½—inhibitor of mitogen-activated protein kinase kinase (MAP2K, MAPK/ERK kinase, or MEK) 1 and 2; ERK ½—extracellular signal-regulated protein kinases 1 and 2; PI3K—phosphoinositide 3-kinase; AKT—protein kinase B; ↓ decrease; ↑ increase.

Due to the plethora of therapeutic properties they are instilled with, natural compounds from the flavonoid class present proven efficacy in CRC treatment through several mechanisms of action. Dietary flavonoids may inhibit tumor formation and act as chemopreventive agents by influencing specific cellular processes causing changes within the colon epithelium. One such effect underlying the biological activity of flavonoids is their inhibitory action on CYP1 through a significant interaction with cytochrome P450 CYP1 enzymes, resulting in the blockage of the initial stages of carcinogenesis [82]. An additional point to mention in this regard is the anti-inflammatory activity of flavonoids, since chronic inflammation constitutes a risk factor in CRC progression. They are also able to activate the antioxidant pathway that potentiates the anti-inflammatory effect by inhibiting the secretion of specific enzymes (e.g., lysozymes or β-glucuronidase) and of arachidonic acid, thus minimizing inflammatory reactions [83]. Some flavonoids (i.e., quercetin) were found to suppress inflammation by downregulating COX2, which is frequently upregulated in CRC [8]. Other antitumor mechanisms characteristic to flavonoids include the induction of apoptosis, inhibition of angiogenesis, modulation of cell signaling pathways, scavenging of oxidative stress, and modulation of the intestinal microbiome [8,82]. Flavonoids also modulate a panel of signaling pathways involved in CRC progression, including NF-kB, Wnt/B-catenin, MAPK and PI3K/AKT/mTOR [8,9]. A schematical representation of several target pathways targeted by flavonoids is presented in Figure 3.

To date, several flavonoids have been subjected to extensive research as potential therapeutics for CRC [8]. For instance, several studies have placed the flavonol quercetin—one of the predominant flavonoids existing in the human diet—at the center of attention for CRC therapy. The work of Temesgen S and collaborators evaluated the antitumor effect of quercetin in the HCT-116 CRC cell line, demonstrating its ability to trigger apoptotic cell death in a dose-dependent manner and inhibit colony formation in both p53-positive and p53-negative cells [84] Another study stressed the antitumor properties of quercetin (25, 50 and 100 µM) in two distinct CRC cell lines, Caco-2 and SW620, by triggering apoptosis in a concentration-dependent manner through nuclear factor kappa-B (NF-kB) inhibition [85]. Similarly, the study conducted by Hashemzaei et al. demonstrated the apoptotic properties of quercetin (10, 20, 40, 80 and 120 µM) in CT-26 CRC cells along with the efficiency of this flavonoid to significantly reduce the volume of CT-29 tumors in BALB/c mice [86]. In addition to these findings, Bathiya et al. demonstrated that the antiproliferative activity of quercetin (80 and 120 µM) in HCT-116, COLO 320 and COLO 205 CRC cells is related to its ability to modulate the expression of anti-aging genes *SIRT-6* and *Klotho* [87].

The anticancer effect of flavolignan silibinin was also widely studied in a variety of CRC cell lines, the results indicating an inhibitory effect on cell growth and proliferation through cell cycle arrest or apoptosis induction [88]. Specifically, a study by Kaur et al. suggested that silibinin shows selectivity towards SW480 CRC cells, inducing cell death and decreasing the nuclear and cytoplasmic levels of β-catenin [89] A previous in vivo study, performed in BALB/c nu/nu male mice presenting HT-29 CRC xenografts, has revealed that silibinin exerts antiproliferative and pro-apoptotic activity by downregulating cyclin D1 expression, Akt phosphorylation and extracellular signal-regulated kinase 1/2 (ERK1/2), while its anti-angiogenic property is strongly correlated with the decrease in vascular endothelial growth factor (VEGF), as well as with the inhibition of cyclooxygenases 1 and 2, hypoxia-inducing factor-1A (HIF-1A), and nitric oxide synthases [90]. Raina and colleagues also found that silibinin altered TNFα-caused NF-κB activation in SW480, LoVo and HT-29 CRC cells, while also downregulating the expressions of NF-κB-regulated molecules (i.e., Bcl-2, COX2, iNOS, VEGF and MMP9) both in CRC cells and mouse xenografts [91].

Naringin is a flavonone that generally acts as an anticancer agent by inducing apoptosis, reducing cell proliferation, modulating different signaling pathways, blocking the cell cycle, inhibiting cell invasion and metastasis, causing autophagy and enhancing cancer cell sensitivity to chemotherapy [92]. Naringin inhibits the PI3k/AKT/mTOR signaling pathway in several cancers, including CRC. In SW620 and HCT116 cells, naringin promoted apoptosis by significantly reducing the expression of anti-apoptotic Bcl-2, and increasing the expression levels of pro-apoptotic Bax [93]. Another finding regarding the antitumor effect of naringin was described by Zeng et al. who demonstrated that naringin promotes apoptosis and inhibits migration in Caco-2, HCT-116, HT-29 and SW480 CRC cells, while also suppressing tumor growth in male nude mice bearing HCT-116 xenografts [94].

The flavonol fisetin proved its anti-CRC potential through in vitro investigation on SW-480 cells by triggering apoptosis and reactive oxygen species production [95]. Another research on the potential applicability of fisetin in CRC therapy has outlined its efficiency in abolishing trypsin-induced cell proliferation, promoting apoptosis, suppressing cell cycle progression and inhibiting cell migration in HCT-116 and HT-29 CRC cells through the modulation of NF-kB pathway. Additionally, the study also concluded that fisetin blocked the growth of HCT-116 tumors in NOD/Shi-scid-IL2R gamma (null) mice [96]. A double-blind randomized placebo-controlled clinical trial has demonstrated the benefits of fisetin as a complementary antineoplastic agent due to its ability to improve the inflammatory status in CRC patients by suppressing the levels of IL-8, hs-CRP and MMP-7 following supplementation [97].

Liu et al. found that chalcone xanthohumol significantly suppressed CRC cell proliferation, colony formation, and xenograft tumor growth, highlighting that the main underlying mechanisms of action are knockout of hexokinase-2, downregulation of glycolysis and activation of intrinsic apoptosis [98]. Another report presented that the anticancer effect of xanthohumol in SW480 CRC cells was related to apoptosis induction, DNA-damage response enhancement and ataxia telangiectasia mutated (ATM) pathway activation [99].

Liang et al. demonstrated that the isoflavones genistein and daidzein exerted their antitumor properties in HT-29 CRC cells by inhibiting the accumulation of lipid droplets and causing apoptosis [100]. Additionally, rutin has also been shown to alter Bax, Bcl-2 and caspase-9 expression levels in HT-29 and Caco-2 CRC cell lines, apigenin increases caspase-3 and Bax expression and decreases Bcl-2 expression in SW480, HCT-116, and HT-29 CRC cell lines and nobiletin has been reported to decrease MMP-7 and PGE2 in HT-29 CRC cells [101].

### 4.2. Flavonoid-Based Nanopharmaceuticals as Potential Therapeutics for CRC

Although many of the currently discovered flavonoids have been described as potential therapeutics with applications in CRC treatment (Section 4.1), their formulation as nanopharmaceuticals using advanced delivery systems that confer additional benefits (i.e., protection against degradation in the bloodstream, targeted delivery, enhanced therapeutic effects, reduced toxicity, improved pharmacodynamic and pharmacokinetic properties) has emerged as a more efficient strategy in the management of this neoplasm, as CRC requires a multidisciplinary treatment approach by integrating new technologies to improve treatment outcomes. In this way, nanotechnology is a tool serving not only in CRC diagnostics, but also in the controlled delivery of bioactive compounds [102]. The most recently developed flavonoid-based nanopharmaceuticals studied for potential CRC therapy are further presented in Table 1.

Additionally, the available preclinical studies demonstrating the efficacy of nanopharmaceuticals based on flavonoids belonging to the flavonone, flavolignan, isoflavone, flavonols, flavone and flavanol classes as CRC therapeutics are detailed below, collectively illustrating their promising applicability in this therapeutic area.

#### 4.2.1. Flavonone Nanopharmaceuticals Studied for Potential CRC Treatment

The formulation of different flavonones as nanopharmaceuticals holds great promise for the development of more effective CRC therapies, as evidenced by recent studies supporting not only the antitumor activity of the respective flavonones when entrapped in different nanocarriers, but also the enhanced therapeutic potential of the obtained nanocomplexes. For instance, Yang et al. investigated the antitumor effect of hesperidin encapsulated in Zn2+@SA/PCT nanocomposites in HCT-116 CRC cells, revealing the efficacy of this formulation in inhibiting cell growth and inducing apoptosis. Cytotoxicity was expressed even at a very low dose of 5 μg/mL, with cell viability reaching approximately 40%, and further decreasing up to 22% at the concentration of 15 μg/mL. The pro-apoptotic properties of this nanopharmaceutical were evidenced by an increase in caspase-9, caspase-3, and Bax expression, as well as by a downregulation of Bcl-2 [103]. In contrast, another study resorted to the encapsulation of hesperidin in poly(lactic-co-glycolic) acid (PLGA) nanoparticles and their in vitro evaluation as potential antitumor agent in HCT-116 CRC cells. The results indicated that empty PLGA nanoparticles did not induce cytotoxicity in HCT-116, while hesperidin alone did not show cell penetrability, having a low capacity and a reduced inhibitory function. Upon encapsulation, however, the anticancer effects of hesperidin were considerably enhanced [126]. The benefits resulting from nanocarrier-based delivery of flavonones were also observed in the case of naringenin, which was loaded into eudragit E100 nanoparticles (NRG-EE100-NPs) and thus evaluated by Sundeep C and co-workers who confirmed an improved therapeutic efficacy after encapsulation, both in vitro and in vivo. Specifically, this optimized naringenin-based nanopharmaceutical showed enhanced cytotoxicity in colon-26 CRC cells, as well as a better suppression of tumor growth in colon-26 tumor-bearing BALB/c mice and a higher survival rate compared to free naringenin. As an additional advantage retained by this nanopharmaceutical, NRG-EE100-NPs demonstrated significantly improved bioavailability after oral administration [104]. Further exploring the potential application of flavonone nanopharmaceuticals in CRC therapy, an additional study has revealed that the incorporation of naringin in pluronic F68-based polymeric micelles leads to a prolonged release compared to the free compound and boosts its antitumor activity against Caco-2 CRC cells [105] and another described the development of a nanostructure containing naringin-reduced graphene oxide nanosheets (rGO@Nar) that showed superior activity in inhibiting HT-29 CRC cells’ growth and inducing apoptosis compared to free naringin when associated with near-infrared (NIR) photothermal therapy [106].

#### 4.2.2. Flavonolignan Nanopharmaceuticals Studied for Potential CRC Treatment

The investigation of flavonolignan-based nanostructures as a promising avenue for CRC treatment was consistently supported by previous studies. A such example is the work of Mombeini et al. who found that silymarin encapsulated in micelles (NANO-SLM) presented an improved efficacy compared to free silymarin, exerting a stronger cytotoxic and pro-apoptotic effects in HT-29 CRC cells [59]. Similar conclusions were also drawn in the case of silibinin, the main compound found in silymarin, which has been previously studied for its anticancer properties on CRC following incorporation in targeted delivery systems. For instance, Shafiei G and associates investigated the antitumor activity of silibinin loaded in magnetic niosome nanoparticles (MNNP) as a potential therapeutic for CRC. The obtained results illustrated that the use of these nanocarriers increases silibinin uptake by HT-29 CRC cells and consequently potentiates its apoptosis-related cellular toxicity [108]. In the same train of ideas, another study conducted by Jackson et al. demonstrated that silibinin-assisted silver nanoparticles (SSNPs) inhibited proliferation, altered morphology, enhanced apoptosis and p53 protein expression in HT-29 CRC cells [107], while the recent work of Rahimnia et al. led to the preparation of a silibinin-containing nanoformulation based on PLGA Nanoparticles decoration with 5TR1 aptamer (SBN-PLGA-5TR1) that exhibited a sustained and constant release of silibinin, a favorable delivery of this flavolignan in acidic CRC environment, selective cytotoxicity in colon-26 and HT-29 CRC cells compared to normal cells, and a pro-apoptotic effect [109]. In addition, another group of researchers has recently assessed the potential applicability of silibinin entrapped in Zein β-cyclodextrin in CRC therapy, illustrating that this complex presented a potent antioxidant activity, along with selective cytotoxicity in HT-29 cells through apoptosis induction demonstrated via caspases-3 and -9 overexpression, NF-κB downregulation and specific apoptotic-like dismorphology of cell nuclei [110].

#### 4.2.3. Isoflavone Nanopharmaceuticals Studied for Potential CRC Treatment

Recent studies revealed the efficacy of several drug delivery systems in improving not only the anti-CRC activity of genistein, but also its release in physiological media and stability against degrading factors. As relevant examples on this matter, genistein encapsulated using bacterial nanocellulose (BNC) and cetyltrimethylammonium-modified BNC (BNC-CTAB) presented controlled and continuous desorption of genistein in gastrointestinal fluids, and antitumor properties in two distinct CRC cell lines, SW480 and SW620, respectively [127], while another paper has confirmed that the encapsulation of genistein in BNC represents a promising strategy to improve the chemoprotective activity of this isoflavone against CRC when administered orally, as this formulation confers protection against gastric acidity and an efficient release in the colonic fluid, which consequently favors a local action on the colonic mucosa, the starting area of the CRC development [128]. Additional to these findings, the study led by Ashok KJ prepared inulin-stearic acid conjugate encapsulating genistein (GNPs) and proved good colloidal dispersibility, high encapsulation efficiency (92%), and potent antiproliferative and apoptotic effects in HCT-116 CRC cells of the synthesized nanocomplex [129]. Another isoflavone, daidzein, has received considerable scientific attention as a promising compound for the potential development of active nanopharmaceuticals in CRC therapy. Sanatkar et al. developed an isoflavone nanopharmaceutical through the loading of daidzein in chitosan nanoparticles (CED) that exhibited concentration-dependent inhibition of HT-29 cells’ proliferation. In addition, signs of apoptosis such as apoptotic body formation, fragmentation of internucleosomal DNA, and chromatin condensation were noticed in these CRC cells treated with this daidzein-based formulation [111]. A mechanistic insight into this subject was given by a recently performed study that revealed the enhanced cytotoxicity of daidzein nanosuspension (DZ-NS) in Caco-2 CRC cells compared to free daidzein, and its ability to improve the chemotherapeutic properties of 5-fluorouracil by increasing p53, decreasing Bcl-2 and suppressing MMP-9 [112].

#### 4.2.4. Flavonol Nanopharmaceuticals Studied for Potential CRC Treatment

Efficient antitumor activity was noticed both in vitro and in vivo after the encapsulation of several flavonols in drug delivery systems. Myricetin, for example, was loaded into solid–lipid nanoparticles (MCN-SLNs) that decreased the percentage of viable cells, triggered apoptosis by increasing Bax expression and decreasing Bcl-2 expression, and generated ROS in HT-29 CRC cells [113], and also incorporated in silver nanoparticles that showed antitumor effects in the HCT-116 CRC cell line [114]. Quercetin was also investigated following entrapment within nanocarriers as a potential CRC therapeutic. Resorting to the functionalization of nanomaterials with polymers, Xu et al. investigated the therapeutic efficacy of quercetin-loaded monomethoxy poly(ethylene glycol)–poly(ε-caprolactone) nanomicelles (Qu-M) in CRC treatment, suggesting that this nanopharmaceutical presents a strong antitumor effect both in vitro and in vivo by causing cytotoxicity and inducing apoptosis in C26 CRC cells, inhibiting tumor growth and proliferation, and blocking angiogenesis in C26 tumor-bearing BALB/c mice more prominently compared to quercetin [115]. A similar observation was made in another study exploring the anticancer properties of quercetin loaded in pH-responsive polymeric nanoparticles. The findings of this research highlighted that the obtained nanostructure exerted an 80-fold increase in potency compared to free quercetin, inhibiting the viability of CT26 CRC cells more prominently [116]. Further supporting the findings related above, an optimized quercetin nanoemulsion using D-tocopheryl polyethylene glycol succinate (TPGS) as a surfactant (QR-NE) demonstrated an enhanced release rate of quercetin and an improved HCT-116 and HT-29 cellular killing efficiency compared to the free flavonoid [117], while cross-linked chitosan nanoparticles (NPs) carrying quercetin exhibited enhanced antiproliferative, pro-apoptotic and anti-angiogenic properties in an in vivo CRC model induced in Wistar rats [118].

The potential benefits of flavonol nanopharmeceutical in CRC management were extended to other flavonols, as well. A study investigating the anti-CRC activity of fisetin encapsulated in polymeric micelles showed that this formulation exhibited a sustained and prolonged release, a more prominent cytotoxicity in CT26 CRC cells by inducing apoptosis, a stronger suppression of tumor progression and a prolonged survival time in CT26 tumor-bearing BALB/c mice compared to free fisetin [114]. A stronger activity was also obtained in the case of rutin, which was more effective in reducing the viability of HT-29 CRC cells when delivered via nanocrystals, this formulation additionally exerted a selective cytotoxicity towards tumor cells [130].

#### 4.2.5. Flavone Nanopharmaceuticals Studied for Potential CRC Treatment

Several therapeutic improvements were obtained through the formulation of some flavones as nanopharmaceuticals. For example, Wu et al. showed that luteolin encapsulated in liposomes (Lipo-Lut) confers a stronger pro-apoptotic effect in CT26 CRC cells and a higher tumor inhibitory potency in CT26 xenograft-bearing BALB/c mice compared to free luteolin [119] Another effective method for the loading of luteolin was employed by Shinde et al. who resorted to biodegradable protein zein nanoparticles as carriers. Their findings revealed that the obtained nanostructures exerted strong radical scavenging activity, high cytotoxicity and pro-apoptotic properties in SW480 CRC cells [120]. Shen and colleagues designed a complex drug delivery system based on aminated MIL-101(Fe) and graphene oxide (GO) containing luteolin and marine (NH2-MIL-101(Fe) @GO@Drugs) with enhanced antitumor activity evidenced through viability reduction, migration inhibition, ROS generation and apoptosis activation via caspase-3 and caspase-9 upregulation in RKO CRC cells [131]. In the case of baicalein, a potentiated therapeutic effect in HT-29 CRC cells was obtained following its delivery via chitosan nanoparticles [132]. In addition to encapsulation in targeted delivery platforms, flavonols were also associated with other therapeutics. One such study was conducted by He Bao and associates who developed and evaluated a combined argynil-glycyl-aspartic acid (RGD)-decorated tangeretin and atorvastatin nanosystem (RGD-ATST/TAGE CNPs) for the treatment of CRC. With a high entrapment efficiency of 90%, this nanosystem caused significant cytotoxicity in HT-29 cells, lacked toxic potential in normal CCD-18 cells, and inhibited tumor development in mice [133]. The research conducted by Banarjee et al. resulted in the obtainment of a stable liposomal nanocarrier containing the hydrophobic flavone apigenin with potential utilization in CRC. The findings of this study revealed that apigenin liposome yields proper hemocompatibility and cytocompatibility in normal fibroblasts, presenting optimal release kinetics and enhanced cytotoxicity in HCT-15 and HT-29 CRC cells compared to free apigenin by inducing G2-M cell cycle arrest. The antitumor properties of this nanopharmaceutical were also confirmed in vivo in athymic nude mice (nu/nu) bearing HT-29 tumor xenografts, as the treatment caused a decrease in tumor volume and vasculature [121]. As regards the impact of nanoencapsulation on the pharmacokinetic properties of flavones, the entrapment of apigenin within whey protein isolate was previously found to improve the overall bioavailability of this compound in mice by increasing its concentrations in plasma and colon mucosa following oral administration. Moreover, this strategy enhanced the cellular uptake of apigenin in HCT-116 and HT-29 CRC cells, exerting pro-apoptotic effects [134]. In another study, Yang et al. designed receptor-selective hyaluronic acid-coated PLGA nanoparticles loaded with apigenin (HA-PLGA-API-NPs) for the specific targeting of CRC presenting a high expression of CD44, demonstrating the improved uptake and strong cytotoxicity in HT-29 CRC cells [122].

#### 4.2.6. Flavanol Nanopharmaceuticals Studied for Potential CRC Treatment

Several research groups have investigated whether the utilization of nanocarriers for the targeted delivery of flavanols brings potential additional benefits to the encapsulated compounds. In this context, Nobahari et al. developed novel nanoformulations by loading catechin into iron oxide nanoparticles coated with sodium alginate and hydroxyapatite and assessed their potential antineoplastic efficacy. The results indicated a time and concentration-dependent decrease in HT-29 cells’ viability and a stronger cytotoxicity compared to the free drug [123]. The recent work of Kassem and collaborators was focused on facilitating a colon-targeted flavanol-based therapy for local diseases such as CRC by developing an intelligent catechin-containing pH-responsive delivery system based on silica nanoparticles coated with Eudragit^®^-S100 that prevented the release of the loaded drug in acidic environment, while ensuring the unloading of approximately 90% of catechin at colonic pH (>7) [135]. In addition to these results, poly(catechin) nanoparticles were also developed as potential treatment strategy for CRC, triggering a strong cytotoxicity in MC38 cells [124]. One study resorted to the encapsulation of another flavanol, epigallocatechin-3-gallate, in pH-sensitive polymeric nanoparticles for CRC treatment, demonstrating an efficient release of this compound at pH of 7.2, an antibacterial activity, and enhanced cytotoxicity against HT-29 CRC cells through apoptosis induction and cell cycle arrest [125].

### 4.3. The Safety Profile of Flavonoids and Flavonoid Nanopharmaceuticals

One of the primary limitations of the current CRC treatments remains the severe toxic events caused by the administered antitumor agents [136], requesting the research of alternative options with enhanced safety profiles and efficient therapeutic activity. A particular feature of flavonoids is that they are characterized by considerable low toxicity compared to conventional chemotherapeutics [51]. Although no toxicity was reported up to 140 g/day [137], some adverse effects such as the disturbance to the intestinal flora, dysregulation of the thyroid function, inhibition of key enzymes involved in hormone metabolism, induction of mutations or generation of oxidative stress might appear when consumed at high doses [138,139]. The potential toxicity caused by flavonoids was also a subject addressed in several in vitro studies that not only reported their lack of cytotoxicity in healthy cell lines, but also a selective effect towards tumor cells [140,141,142].

As regards the evaluation of flavonoid nanopharmaceuticals, their potential application in CRC treatment was extensively assessed in previous studies, as presented in Section 4.2. Nonetheless, considering the interest in their potential inclusion in the next-generation CRC therapy, their biocompatibility and safety should be also thoroughly addressed. Among the developed nanocarriers encapsulating flavonoids for potential CRC management, some were also explored in terms of potential toxicity and the obtained results were promising. For instance, silymarin-loaded nanoparticles expressed no noticeable effect on the viability and proliferation of NIH-3T3 fibroblasts, while displaying a toxic action only on CRC cells [59], and silibinin-loaded magnetic niosomal nanoparticles showed increased biocompatibility in HEK-293 healthy cells following treatment [108]. Similarly, daidzein-loaded chitosan microcapsules were found to lack toxicity in HDF fibroblasts, causing changes only in the proliferation of HT-29 CRC cells [111]. In addition to these in vitro evaluations, some nanocomplexes directed towards CRC therapy containing naringenin and fisetin presented an increased survival rate after treatment in vivo compared to the free flavonoids [104,114]. All in all, the studies conducted up to date on the biosafety of flavonoids and their nanoformulations are promising, indicating proper biocompatibility and low risk for severe side effects.

## 5. Conclusions and Future Perspectives

CRC remains one of the most pressing global health issues at present by significantly contributing to the overall cancer-related mortality rates and presenting inefficient treatment options due to developed resistance or severe adverse effects. Flavonoids have become one of the most bioactive phytocompounds for CRC treatment, exhibiting multifaceted activities and targeting multiple key pathways that halt tumor development, growth and invasion. As a consequence of their innate but improper features such as hydrophobic nature and insolubility in aqueous environments, poor permeability and low bioavailability that hinder the potential utilization in CRC management, an innovative trend resorting to the tumor-targeted delivery of flavonoids as nanopharmaceuticals obtained through the encapsulation within distinct nanoscaled carriers that not only subside the limitations of the entrapped agents, but also augment their biological effects, has emerged, leading to potential improved CRC treatment outcomes. The present work conferred a mechanistic review of the anti-CRC properties retained by various flavonoid structures, and provided an overview of the most recent findings regarding the targeted CRC treatment using flavonoid-based nanopharmaceuticals. Apart from the general conclusion that drug delivery nanosystems stand as the most futuristic approach allowing the efficient and successful application of all flavonoid classes in CRC therapy despite their differential chemical structures, this review also highlights in the end the importance of the proper selection of the most relevant carrier depending on the administration route (i.e., oral or parenteral), specific flavonoid physicochemical properties (i.e., stability in gastrointestinal fluids), and therapeutic goals (i.e., sustained release).

As future perspectives, several state-of-the-art ideas that could bring additional benefits in the exploration of flavonoids in CRC and enlarge this scientific domain have been identified: (i) de novo and CRC-targeted design of semisynthetic flavonoid derivatives based on deep quantitative structure–activity relationship (QSAR) and generative modeling [143]; (ii) artificial intelligence (AI)-enabled virtual target identification and screening of the most efficient flavonoids and derivatives for CRC [144]; and (iii) AI-driven development and optimization of drug delivery systems for flavonoid encapsulation in CRC [145]. Additionally, although some nanocarriers (e.g., polymer nanoparticles and carbon nanoparticles) and flavonoids (e.g., genistein) have reached clinical trials for potential utilization in CRC treatment [146], to the best of our knowledge, flavonoid nanopharmaceuticals lack clinical investigations that should be conducted in future studies to reinforce their safety and efficacy for CRC therapy.

## Figures and Tables

**Table 1:** Representative studies outlining the antitumor effect of flavonoid-based nanopharmaceuticals as potential therapeutics for colorectal cancer (CRC).

Class	Flavonoid	Type of Delivery System	Flavonoid Encapsulation/Loading or Release Efficacy	Experimental Model for CRC	Tested Concentration/Dose	Anti-CRC Activity	Ref.
**Flavonones**	Hesperidin	Zinc cross-linked nanopolymer blend	EE = 62.8%	HCT-116 cells	0–15 μg/mL	↓ cell viability and growth ↑ ROS generation ↑ apoptosis through caspase-3, caspase-9, cleaved PARP, Bax upregulation and Bcl-2 downregulation	[103]
	Naringenin	Cationic-polymeric nanoparticles	EE = 68.83 ± 3.45%	Colon-26 cells	0.1–100 µg/mL	↓ cell growth	[104]
				Colon-26 tumor-bearing BALB/c mice	40 mg/kg	↓ tumor growth and weight ↑ the survival rate with 50% compared to free naringenin	
		Polymeric micelles	EE = 96.14% ± 2.29 LE = 1.89 ± 0.05	Caco-2 cells	IC_50_ = 0.19 ± 0.01 μM	↑ cytotoxicity	[105]
	Naringin	Graphene oxide nanosheets	n/a	HT-29 cells	0.39–12.5 μM	↑ cytotoxicity ↑ apoptosis evidenced by apoptotic-like morphological features	[106]
**Flavolignans**	Silymarin	Nanomicelles	EE = 99.48%	HT-29 cells	25 μM/mL	↓ cell viability and colony numbers ↑ early and late apoptosis	[59]
	Silibinin	Silver nanoparticles	n/a	HT-29 cells	2–10 ng/mL	↓ cell proliferation ↑ apoptosis and p53 expression	[107]
		Magnetic niosome nanoparticles	LE = 90% RE = 49% (pH = 7.4) and 70% (pH = 5.8)	HT-29 cells	10–80 μg/mL	↓ cell viability ↑ apoptosis	[108]
		PLGA nanoparticles decorated with 5TR1 aptamer	EE = 70.19 ± 1.63%	Colon-26 and HT-29 cells	10–500 μM	↑ the antiproliferative effect ↓ cell viability ↑ apoptosis	[109]
		Zein-β cyclodextrin nanocarrier	LE = 87.25% RE = 40.75%	HT-29 cells	7.8–500 µg/mL	↓ cell viability ↑ apoptosis through caspase-3 and caspase-9 activatioN	[110]
**Isoflavones**	Daidzein	Chitosan nanoparticles	EE = 64.33%	HT-29 and	12.5–100 μg/mL	↓ cell viability ↑ apoptosis through caspase-3 activation	[111]
		Nanosuspension	n/a	Caco-2 cells	7.81–2000 µM	↑ cytotoxicity ↓ proliferation and inflammation ↑ chemotherapeutic efficacy of 5-FU ↑ apoptosis ↑ p53 expression ↓ Bcl-2, IL-6, TNF-α, MMP-9 levels	[112]
**Flavonols**	Myricetin	Solid–lipid nanoparticles	EE = 90%	HT-29 cells	30 μM	↓ cell viability and colony number ↑ apoptosis ↑ Bax, AIF and ROS levels ↓ Bcl-2 and MMP expressions	[113]
	Fisetin	Polymeric micelles	LE = 9.88 ± 0.14% EE = 98.53 ± 0.02%	CT26 cells	0–100 µg/mL	↑ cytotoxicity and cellular uptake ↑ apoptosis	[114]
				CT26 tumor-bearing BALB/c mice	50 mg/kg	↑ survival time sustained and prolonged release ↓ tumor progression	
	Quercetin	Poly(ethylene glycol)–poly(ε-caprolactone) nanomicelles	EE = 97.8% RE = 81.9%	CT26 cells	0–35 µg/mL	↓ cell growth ↑ apoptosis	[115]
				CT26 tumor-bearing BALB/c mice	50 mg/kg	↓ tumor growth and proliferation ↓ angiogenesis	
		pH-sensitive polymeric nanoparticles	EE = 41.8%	CT26 cells	0.1–1000 μM	↓ cell viability	[116]
		Nanoemulsion	EE > 80% RE = 84.52 ± 0.71%	HCT-116 and HT-29 cells	5–100 µM	↓ cell viability	[117]
		Chitosan nanoparticles	LE = 96%	Wistar rats with induced colon cancer	3 mL	↑ antiproliferative, pro-apoptotic and anti-angiogenic properties	[118]
**Flavones**	Luteolin	Liposomes	EE = 90% RE = 80%	CT26 cells	0–30 µg/mL	↓ cell viability ↑ apoptosis	[119]
				CT26 tumor-bearing BALB/c mice	50 mg/kg	↓ tumor vascularization ↓ tumor volume and weight ↓ Ki-67 expression ↑ apoptosis	
		Zein nanoparticles	EE = 92%	SW480 cells	n/a	↓ cell viability ↑ cellular uptake ↑ apoptosis	[120]
	Apigenin	Liposomes	EE = 90%	HCT-15 and HT-29 cells	1.5625–200 μM	↓ cell viability induction of G2-M cell cycle arrest ↑ ATM, p53 and p21 expressions ↓ Cyclin B1 expression	[121]
				HT-29 tumor-bearing athymic nude mice	50 mg/kg	↓ in tumor volume and vasculature ↓ CD-31 and Ki-67 expressions	
		Hyaluronic acid-coated PLGA nanoparticles	EE = 90.56% ± 0.57 LE = 3.19% ± 0.03	HT-29 cells	0–150 μg/mL	↓ cell viability	[122]
				HT-29 tumor-bearing nude mice	n/a	↑ accumulation at the tumor site	
**Flavanols**	Catechin	Iron oxide nanoparticles coated with sodium alginate and hydroxyapatite	EE = 81.25 ± 2.55% LE = 20.31 ± 0.64%	HT-29 cells	50–500 µg/mL	↓ cell viability ↑ apoptosis	[123]
		Degradable poly(catechin) nanoparticles	n/a	MC38 cells	0–1000 µg/mL	↓ cell viability	[124]
	Epigallocatechin-3-Gallate	pH-sensitive polymeric nanoparticles	EE and RE > 93%	HT-29 cells	n/a	↑ cytotoxicity ↑ apoptosis and cell cycle arrest	[125]

↑ increase; ↓ decrease; CRC—colorectal cancer; ROS—reactive oxygen species; PARP—poly (ADP-ribose) polymerase; p53—protein p53; 5-FU—5-fluorouracil; IL-6—interleukin 6; TNF-α—tumor necrosis factor; MMP-9—matrix metalloproteinase-9; AIF—apoptosis-inducing factor; Ki-67—antigen Kiel 67, protein; ATM—ataxia telangiectasia–mutated gene; p21—protein p21; CD-31—platelet/endothelial cell adhesion molecule-1; EE—encapsulation efficacy; LE—loading efficacy; RE—release efficacy; n/a—not available; Ref—reference.

## Data Availability

Not applicable.
